# Decomposition of Disparities in Alzheimer’s Disease and Related Dementia

**DOI:** 10.1093/geroni/igab046.1063

**Published:** 2021-12-17

**Authors:** Igor Akushevich

**Affiliations:** Duke University, Durham, North Carolina, United States

## Abstract

This study uses Medicare data to non-parametrically evaluate race- and place-of-residence-related disparities in AD/ADRD prevalence and incidence-based mortality, separate them out into the epidemiological causal components including race-related disparities in incidence and survival, and finally explain these in terms of health-care-related factors using causal methods of group variable effects (propensity scores and the rank-and-replace method) and regression-based analyses (extended Fairlie’s model and generalized Oaxaca-Blinder approach for censoring outcomes). Partitioning analysis showed that the incidence rate is the main predictor for temporal changes and racial disparities in AD/ADRD prevalence and mortality, though survival began to play a role after 2010. Arterial hypertension is the leading predictor responsible for racial disparities in AD/ADRD risks. This study demonstrated that Medicare data has sufficient statistical power and potential for studying disparities in AD/ADRD in three interacting directions: multi-ethnic structure of population, place of residence, and time period.

